# Circulating miRNAs miR-34a and miR-150 associated with colorectal cancer progression

**DOI:** 10.1186/s12885-015-1327-5

**Published:** 2015-04-30

**Authors:** Sinéad T Aherne, Stephen F Madden, David J Hughes, Barbara Pardini, Alessio Naccarati, Miroslav Levy, Pavel Vodicka, Paul Neary, Paul Dowling, Martin Clynes

**Affiliations:** 1 Molecular Therapeutics for Cancer Ireland, National Institute for Cellular Biotechnology, Dublin City University, Glasnevin, Dublin 9, Ireland; 2 Department of Physiology and Medical Physics and Centre for Systems Medicine, Royal College of Surgeons in Ireland, Dublin 2, Ireland; 3 Human Genetics Foundation, Turin, Italy; 4 1st Medical Faculty of Charles University and Thomayer Hospital, Prague, Czech Republic; 5 Institute of Experimental Medicine, Academy of Sciences of the Czech Republic, Prague, Czech Republic; 6 Department of Colorectal Surgery, AMNCH Hospital, Dublin 24, Ireland; 7 Department of Biology, Maynooth University, Maynooth Co., Maynooth, Co. Kildare Ireland

**Keywords:** Colorectal cancer, Circulating miRNAs, miR-34a, miR-150, miR-923

## Abstract

**Background:**

Screening for the early detection of colorectal cancer is important to improve patient survival. The aim of this study was to investigate the potential of circulating cell-free miRNAs as biomarkers of CRC, and their efficiency at delineating patients with polyps and benign adenomas from normal and cancer patient groups.

**Methods:**

The expression of 667 miRNAs was assessed in a discovery set of 48 plasma samples comprising normal, polyp, adenoma, and early and advanced cancer samples. Three miRNAs (miR-34a, miR-150, and miR-923) were further examined in a validation cohort of 97 subjects divided into the same five groups, and in an independent public dataset of 40 CRC samples and paired normal tissues.

**Results:**

High levels of circulating miR-34a and low miR-150 levels distinguished groups of patients with polyps from those with advanced cancer (AUC = 0.904), and low circulating miR-150 levels separated patients with adenomas from those with advanced cancer (AUC = 0.875). In addition, the altered expression of miR-34a and miR-150 in an independent public dataset of forty CRC samples and paired normal tissues was confirmed.

**Conclusion:**

We identified two circulating miRNAs capable of distinguishing patient groups with different diseases of the colon from each other, and patients with advanced cancer from benign disease groups.

**Electronic supplementary material:**

The online version of this article (doi:10.1186/s12885-015-1327-5) contains supplementary material, which is available to authorized users.

## Background

Colorectal cancer (CRC) poses a significant threat to the health of global populations; it is the second most commonly diagnosed cancer in females and the third in males [1]. CRC develops in a progressive fashion during which normal colon epithelial cells transform to form benign growths such as polyps. These polyps may then progress to benign adenomas, and ultimately to invasive cancer lesions. The progression of the cancer has also been associated with sequential genetic changes in genes such as *K-RAS, APC, DCC,* and *P53* [2]. However CRC is a heterogeneous disease with various patient-related confounding factors such as the anatomic location of the tumour, race/ethnicity of the patient, and genetic and dietary interactions influencing the development of the disease [3].

Screening at risk populations for CRC has significantly improved the outcome for patients, for instance diagnosis while the disease remains localised to the colon dramatically improves patient survival, and removal of early lesions such as adenomatous polyps may prevent disease formation [4]. There are currently several potential screening tests available to detect CRC including the faecal occult blood test (FOBT), flexible sigmoidoscopy (FS), optical colonoscopy (OC) and computed tomography colonography (CTC). FOBT is a simple, cheap and safe test that relies on the assumption that large adenomas and cancerous lesions may bleed, and that these blood products are detectable in the faecal matter of patients. Although cheap and non-invasive, this test is vulnerable to false positive and negative results due to incorrect sample storage, or confounding medical complaints such as haemorrhoids. The other examinations involve more costly and invasive procedures which although allow direct access to colorectal lesions also suffer from low patient acceptance and procedural risks such as perforation of the colon [4].

The focus of the scientific community has thus shifted to exploring the identification of non-invasive biomarkers of disease from bio-fluids such as saliva, urine, and blood. MicroRNAs (miRNAs) are nucleic acid markers that have been recently investigated in this context. MiRNAs are short (20-22nt) non-coding RNAs that negatively regulate gene expression through either mRNA degradation or translational repression [5]. MiRNA expression has been shown to be altered in cancerous tissue compared to normal tissue and different miRNAs have been attributed oncogenic and tumour suppressor qualities [6]. In 2008, Chen *et al.* detected miRNAs in the serum and plasma blood components of humans and other animals. This primary study illustrated that miRNAs remain stable in serum after being subject to severe conditions such as extremely low or high pH, 10 freeze-thaw cycles, extended storage, boiling, and RNase digestion [7]. In addition to their presence in serum and plasma, miRNAs have also been detected in other body fluids such as urine, saliva, and amniotic fluid making them ideal potential candidates as non-invasive biomarkers of disease [8].

Expression levels of circulating miRNAs have shown some potential at distinguishing cancer patients and healthy controls for prostate [9], ovarian [10], lung [11,12], and breast cancers [13]. Several studies have also investigated circulating miRNA levels for the detection of CRC. Initial approaches analysed small numbers of circulating miRNAs in CRC patient samples compared to normal controls [14]. Other groups performed miRNA profiling on pooled plasma samples and validated candidate biomarkers on additional individual samples [15], and others performed profiling on a small number of CRC tissue/serum/plasma samples before validation in a larger sample set [16]. These studies have produced conflicting results [17] and so recently, groups have begun to perform profiling on larger sample sets and included plasma from patients with adenomas in addition to CRC to improve the specificity of disease detection [18].

In 2008, a guideline was released from the American Cancer Society which highlighted the importance for patients to have access to screening tests that will facilitate cancer prevention through the early detection of cancer, and the detection and removal of polyps [4]. A clear deficit in the search for circulating biomarkers for the early detection of CRC to date is the lack of adenomatous polyp samples and the lack of separation of advanced and early stage cancers represented in studies [14-16,18-20]. The aim of this study was therefore to investigate the potential of circulating cell-free miRNAs not only as biomarkers of CRC, but also their efficiency at delineating patients presenting with polyps and benign adenomas from normal and cancer groups. To facilitate this we performed miRNA profiling for 667 miRNAs on a discovery set of 48 plasma samples comprising 8 normal, 8 polyp, 16 adenoma samples, 8 early stage cancer samples (stage I/II), and 8 advanced cancer samples (stage III/IV). Three candidate miRNAs; miR-34a, miR-150, and miR-923 were then further examined in a validation cohort of 97 independent plasma samples comprising 20 normal, 20 polyp, 20 adenoma samples, 23 early stage cancer samples, and 14 advanced cancer samples. In addition, we confirmed the altered expression of two of the miRNAs in an independent dataset of 40 CRC samples and their paired normal tissues. We found circulating levels of miR-34a and miR-150 to be capable of distinguishing patients groups with benign and malignant diseases of the colon from each other, and sets of miRNAs that distinguish patients with advanced cancer from benign disease groups. Specifically, we found high levels of circulating miR-34a and low miR-150 levels to distinguish patients with polyps from those with advanced cancer, and low circulating miR-150 levels to separate patients with adenomas from those with advanced cancer.

## Methods

### Patients selection and sample collection

Cases with positive colonoscopy results for malignancy, confirmed by histology as colon or rectal carcinomas, were recruited between December 2007 and December 2010 at the Department of Surgery, Adelaide and Meath Hospital and at the Thomayer Hospital in Prague, Czech Republic. Control subjects or subjects diagnosed with polyps or adenomatous polyps were selected during the same period from individuals undergoing colonoscopy for various gastrointestinal complaints (macroscopic bleeding, positive faecal occult blood test or abdominal pain of unknown origin). The participating subjects gave written informed consent in accordance with the Declaration of Helsinki at the precipitating site that was approved by Tallaght Hospital/St. James’s Hospital Joint Research Ethics Committee, The Adelaide and Meath Hospital, Dublin, Incorporating The National Children’s Hospital, Tallaght, Dublin 24, Ireland and the Ethical Committee of the Institute of Experimental Medicine, Prague, Czech Republic. See Table 1 for clinical information on samples used.

**Table 1:** **Clinical information on the discovery and validation plasma sample cohorts**

Discovery Cohort		
	n (M/F)	Age
**Normal**	8 (4/4)	67 ± 11
**Polyps**	8 (4/4)	65 ± 7
**Adenoma**	16 (8/8)	56 ± 6
**Early Stage Cancer (Stage I/II)**	8 (4/4)	65 ± 10
**Advanced Cancer (Stage III/IV)**	8 (4/4)	68 ± 8
**Validation Cohort**		
	**n (M/F)**	**Age**
**Normal**	20 (12/8)	63 ± 8
**Polyps**	20 (11/9)	57 ± 7
**Adenoma**	20 (12/8)	62 ± 10
**Early Stage Cancer (Stage I/II)**	23 (10/13)	63 ± 12
**Advanced Cancer (Stage III/IV)**	14 (9/5)	67 ± 8

M denotes male; F denotes female.

Two separate patient cohorts were identified, a discovery set (n = 48) comprising 8 normal, 8 polyp, 16 adenoma samples, 8 early stage cancer samples (stage I/II), and 8 advanced cancer samples (stage III/IV), and a validation set (n = 97) comprising 20 normal, 20 polyp, 20 adenoma samples, 23 early stage cancer samples, and 14 advanced cancer samples. In addition, an independent public dataset [21] of quantitative real-time PCR (qRT-PCR) raw data was downloaded from the NCBI GEO archive (accession no: GSE28364) which contains information on 40 CRC samples and their paired normal tissues.

Plasma samples were collected according to standard phlebotomy procedures. 10 ml of blood sample was collected into EDTA plasma tubes and immediately placed in ice. The tubes were centrifuged at 1000 x g for 10 minutes at 4°C. Plasma was denuded by pipette from the cellular material, aliquoted into cryovial tubes, labelled and stored at -80°C until the time of analysis. The time from sample procurement to storage at -80°C was less than 3 hours. Each plasma sample underwent no more than 3 freeze/thaw cycles prior to analysis.

### RNA extraction

Total RNA was isolated from 60 μl of each plasma sample using the miRNeasy mini kit (Cat no 217004, Qiagen). The Qiagen supplementary protocol (Purification of total RNA, including small RNAs, from serum or plasma) was utilised with the following modifications: thawed plasma samples were centrifuged at 1000 x g for 5 minutes at 4°C to remove excess debris from samples, RNA was extracted from the upper 50 μl of each sample. To elute the RNA, 50 μl of nuclease-free water was added to each spin column and incubated for 1 minute at room temperature before centrifuging into non-stick RNase-free microfuge tubes (Cat no AM12350, Ambion) to elute the RNA.

### MiRNA profiling of plasma with TaqMan® low-density arrays

TaqMan® Array Human MicroRNA A and B Cards v2.0 (Cat no 4400238, Applied Biosystems) were employed to examine the expression of 667 miRNAs in 48 plasma samples in the discovery cohort. Reverse transcription and quantitative PCR (qPCR) were performed on equal volumes of RNA from each sample according to the manufacturer’s instructions using TaqMan® MicroRNA Reverse Transcription Kit (Cat no 4366596, Applied Biosystems) and Megaplex RT Primers to convert the miRNAs to cDNA, TaqMan® PreAmp Master Mix (Cat no 4391128, Applied Biosystems) and Megaplex PreAmp Primers for a preamplification step before real-time analysis. qPCR was performed using TaqMan® Universal Master Mix II, no UNG (Cat no 4440048, Applied Biosystems) on the 7900HT Fast Real-Time PCR system (Applied Biosystems). The Sequence Detector System software version 2.2.2 was utilised to generate study files using a fixed threshold value of 0.1 for statistical analysis (accession no: GSE67075).

### Validation of miRNA expression using qRT-PCR

Individual TaqMan® miRNA assays were used for miRNA quantification in the 97 plasma samples in the validation cohort. To improve reverse transcription efficiency a miRNA multiplex RT primer pool was made from the singleplex RT primers of the four miRNAs to be analysed; miR-34a, miR-150, miR-923, and miR-let7e (this miRNA was used as the endogenous control as it showed very little variation in the discovery cohort, ΔC_t_ SD = 0.865). 100 μl of each 20X RT primer were added to an RNase-free microfuge tube. The tube was dried in a speed vacuum (MAXI dry plus, Medical Supply Company, Ireland) at 50°C for 1 hour. The primers were re-suspended in 100 μl of nuclease-free water and 300 μl of 0.1X TE buffer was added to yield a 5X multiplex RT primer pool. The TaqMan® MicroRNA Reverse Transcription Kit (Cat no 4366596, Applied Biosystems) was used to perform reverse transcription reactions. Each reaction contained 1.8 μl of RT buffer (10X), 0.18 μl of dNTPs (25 mM), 3.6 μl of miRNA multiplex RT primer pool (5X), 1.2 μl of Multiscribe RT enzyme (50 U/μl), 5.22 μl of nuclease-free water and 6 μl of extracted total RNA. The reactions were incubated at 16°C for 30 minutes, 42°C for 30 minutes and 85°C for 5 minutes (G-STORM, GS1, Somerton Biotechnology Centre, UK).

Real-time PCR analysis was performed on 96 well plates (Cat no 4346906, Applied Biosystems). Technical triplicate PCRs were performed for each sample, and no template controls and a pooled sample containing cDNA from all 97 samples were included on each plate to ensure inter-plate reproducibility. Each reaction contained 1 μl of TaqMan miRNA assay (20X), 10 μl of TaqMan® Universal Master Mix II, no UNG (Cat no 4440048, Applied Biosystems), 7.67 μl of nuclease-free water, and 1.33 μl of cDNA. The reactions were incubated at 95°C for 10 minutes, and 40 cycles of 95°C for 15 seconds and 60°C for 15 seconds on the 7900HT Fast Real-Time PCR system (Applied Biosystems). The Sequence Detector System software version 2.2.2 was utilised to generate study files using a fixed threshold value of 0.1 for statistical analysis.

### Statistical analysis

In the discovery cohort (n = 48), each miRNA was normalised by the ΔΔC_t_ method using the average within sample C_t_ value [22]. This technique involves the use of the mean expression value of all expressed microRNAs in a given sample as a normalisation factor for microRNA real-time quantitative PCR data. Thus the average within sample C_t_ value for each card is calculated by averaging all miRNA Ct values for each individual sample. This was performed using the Bioconductor package HTqPCR (www.bioconductor.org). The non-parametric Kruskal-Wallis test was used to determine between group variations by rank as the data was not normally distributed. A Wilcoxon rank sum test was subsequently used to perform pair-wise comparisons between the 5 groups for the significant miRNAs identified by the Kruskal-Wallis test.

As an alternative to spiking un-related miRNA constructs into our samples we utilised the miRNA profiling data of the discovery cohort of samples to choose an appropriate endogenous control for use in the validation cohort. This involved analysing the expression of all 667 miRNAs across all 48 samples in the discovery cohort allowing us to choose one of the least variant miRNAs. As MammU6 showed highly variant expression in the discovery cohort, miR-let7e was chosen for use as an endogenous control for the validation set as it was one of the least variant miRNAs in the discovery phase experiment (ΔC_t_ standard deviation of 0.86). When the let7e C_t_s were examined across all samples in the validation cohort this miRNA proved an appropriate endogenous control with a C_t_ standard deviation of 1.64. Statistically significant differences were determined using the non-parametric Wilcoxon rank sum test. The p-values for the validation set were adjusted using the Benjamini and Hochberg method [23] to account for multiple testing.

For consistency, the independent public dataset from Reid *et al*. [21] (accession no: GSE28364) was normalised using the same approach used to analyse the discovery cohort qRT-PCR data. This independent study used TaqMan® Array Human MicroRNA Cards v2.0 to analyse miRNA expression in 40 CRC tumour samples and their paired normal tissues. In order to mimic this structure in our validation plasma sample cohort, we grouped samples into ‘non-malignant’ and ‘malignant’ groups. As there were only two groups (normal versus cancer) in this analysis, the Wilcoxon rank sum test was used to determine significantly differentially regulated miRNAs. For this analysis of the validation cohort, miR-34a, miR-150 and miR-923 were first normalised against the endogenous control (miR-let7e) and the Wilcoxon rank sum test was used to determine significance between the groups.

Logistic regression (LR) and receiver operator characteristic (ROC) curve analysis were performed on miR-34a, miR-150 and miR-923 in the validation cohort. The markers were combined using LR and the ROC curves were used for interpretation of the models generated. The area under the curve (AUC) from the ROC curve for a given model was used to determine the probability of a correct prediction. The LR model for single miRNAs or combinations of miRNAs which gave the highest AUC was considered the most discriminating model and therefore the best marker at distinguishing between the groups of interest. All calculations were carried out in the R statistical environment (http://cran.r-project.org/) using the HTqPCR and stats packages.

## Results

### Differential expression of miRNAs in the discovery cohort

This study examined the expression of 667 miRNAs in plasma samples of a discovery cohort of 48 patients with benign and malignant disease of the colon compared to age and sex matched disease-free controls (Table 1). Statistical analysis revealed 73 miRNAs that have significantly different levels (p-value <0.05) in at least one of the disease groups (polyp n = 8, adenoma n = 16, early stage cancer (stage I/II) n = 8, and advanced cancer (stage III/IV) n = 8) compared to the healthy controls n = 8 (Table 2). Forty miRNAs were significantly different in advanced cancer, 22 in early stage cancer, 7 in adenoma, and 22 in the polyp group compared to normal controls. Ten miRNAs were significantly altered in both the early stage and advanced cancer groups compared to the normal controls; miR-923, miR-801, miR-144*, miR-135a*, miR-500, miR-497, miR-150, miR-30c and RNU48 showed lower levels while miR-532-3p was more abundant in the cancer groups compared to the controls. MiR-34a was significantly higher in early stage cancer compared to healthy controls.

**Table 2:** **miRNAs with significantly different levels in disease groups compared to normal in the discovery cohort**

MiRNAs	Normal vs Polyp		Normal vs Adenoma		Normal vs Early Cancer		Normal vs Late Cancer	
	Log_2_FC	P Value	Log_2_FC	P Value	Log_2_FC	P Value	Log_2_FC	P Value
hsa-let-7b	−0.823	0.007	−0.618	0.038	−0.712	0.083	−0.298	0.328
hsa-let-7c	3.407	0.442	4.27	0.136	4.794	0.105	0.812	0.753
hsa-let-7g	−0.749	0.195	−0.231	0.153	−1.177	0.010	−3.933	0.234
hsa-miR-135a	13.518	0.050	0.614	0.968	−5.76	0.171	3.738	0.655
hsa-miR-140-5p	0.672	0.038	0.122	0.834	0.558	0.083	−2.645	0.021
hsa-miR-146b-3p	11.496	0.281	−1.962	0.511	−1.377	0.904	10.34	0.043
**hsa-miR-150**	**−0.523**	**0.161**	**−0.706**	**0.032**	**−1.491**	**0.015**	**−2.771**	**0.000**
hsa-miR-15b	−0.538	0.130	−0.362	0.238	−2.019	0.001	−0.746	0.105
hsa-miR-182	7.054	0.178	−2.384	0.864	−3.173	0.685	−11.924	0.032
hsa-miR-183	−0.192	0.599	−1.533	0.667	−3.09	0.792	−15.707	0.010
hsa-miR-190	−0.139	0.874	−9.03	0.043	−16.68	0.018	−7.941	0.075
hsa-miR-191	0.138	0.505	−0.049	0.383	−0.979	0.083	0.509	0.279
hsa-miR-192	3.363	0.721	2.913	0.061	3.349	0.505	−2.113	0.036
hsa-miR-193a-5p	4.76	0.105	2.668	0.417	3.744	0.798	−1.73	0.012
hsa-miR-194	2.184	0.105	2.992	0.452	3.008	0.234	−1.904	0.016
hsa-miR-199a-3p	1.037	1.000	−0.095	0.264	−0.217	0.234	−2.353	0.010
hsa-miR-19a	1.229	0.050	0.318	0.976	1.58	0.028	0.831	0.083
hsa-miR-19b	1.125	0.010	0.302	0.976	1.447	0.065	0.696	0.028
hsa-miR-204	12.069	0.005	6.087	0.096	6.565	0.751	3.338	0.790
hsa-miR-210	2.864	0.003	−3.207	0.927	1.987	0.028	−1.692	0.105
hsa-miR-21	−0.232	0.050	−0.239	0.238	0.561	0.130	−3.683	0.065
hsa-miR-219-1-3p	−2.985	0.382	−1.557	0.610	3.221	0.488	8.939	0.142
hsa-miR-23a	3.751	0.574	−1.265	0.667	0.948	0.400	3.146	0.012
hsa-miR-25	0.717	0.721	0.052	0.120	0.853	0.574	−0.621	0.038
hsa-miR-30b	0.04	0.721	−0.117	0.452	−0.964	0.028	−0.88	0.130
hsa-miR-30c	−0.017	0.878	−0.231	0.172	−0.845	0.038	−6.451	0.038
hsa-miR-323-3p	−0.461	0.328	0.328	0.452	0.243	0.574	−2.93	0.130
hsa-miR-337-5p	6.448	0.359	4.019	0.391	3.964	0.547	12.303	0.007
**hsa-miR-34a**	**18.817**	**0.003**	**12.09**	**0.119**	**15.428**	**0.018**	**11.626**	**0.055**
hsa-miR-365	0.257	0.878	−1.16	0.061	−0.884	0.279	−5.763	0.010
hsa-miR-370	9.181	0.040	4.218	0.283	−2.578	0.790	6.114	0.065
hsa-miR-377	0	NA	4.206	0.221	3.063	0.382	14.295	0.013
hsa-miR-451	0.216	1.000	−0.341	0.120	−0.111	0.721	−1.955	0.007
hsa-miR-486-3p	−0.225	0.382	0.062	0.569	0.176	0.574	−5.158	0.010
hsa-miR-486-5p	−0.813	0.065	−0.241	0.787	−0.562	0.798	−2.187	0.003
hsa-miR-500	−5.223	0.018	−6.169	0.084	−11.283	0.007	−9.256	0.011
hsa-miR-503	−3.035	0.382	−1.631	0.610	−3.035	0.382	7.943	0.225
hsa-miR-532-3p	2.845	0.195	3.061	0.452	1.402	0.015	2.436	0.050
hsa-miR-532-5p	4.749	0.105	3.772	0.787	4.688	0.130	0.31	0.834
hsa-miR-542-3p	3.213	0.382	0	NA	0	NA	7.988	0.076
hsa-miR-548d-3p	−3.186	0.382	−0.077	0.958	2.882	0.700	12.243	0.117
hsa-miR-548d-5p	9.445	0.076	1.349	0.536	2.736	0.382	14.573	0.013
hsa-miR-654-3p	10.564	0.012	4.161	0.153	3.815	0.250	11.441	0.007
hsa-miR-660	0.544	0.234	0.228	0.106	0.447	0.721	−4.865	0.007
RNU48	−3.549	0.365	1.637	0.878	−12.871	0.064	−7.855	0.075
hsa-let-7a-3p	10.399	0.148	8.684	0.217	−6.667	0.171	10.717	0.118
hsa-miR-135-3p	−0.835	0.195	−0.512	0.417	−1.688	0.002	−2.27	0.001
hsa-miR-136-3p	5.14	0.028	−0.617	0.357	−0.031	1.000	2.942	0.016
hsa-miR-138-1-3p	−0.353	0.065	−0.038	0.697	−0.033	0.721	−1.37	0.038
hsa-miR-144-5p	−1.373	0.065	−1.208	0.011	−3.164	0.001	−2.299	0.028
hsa-miR-151-3p	0.398	0.574	0.343	0.697	0.623	0.574	2.57	0.007
hsa-miR-16-1-3p	2.883	0.873	−7.605	0.117	7.397	0.290	4.03	0.424
hsa-miR-221-5p	−6.94	0.171	1.828	0.732	−6.94	0.171	10.556	0.183
hsa-miR-222-5p	3.311	0.566	−3.168	0.479	−2.906	0.590	12.137	0.104
hsa-miR-25-5p	18.99	0.018	5.097	0.334	17.026	0.024	12.007	0.117
hsa-miR-30a-3p	0.171	0.382	−0.048	0.881	0.736	0.003	−3.516	0.279
hsa-miR-30e-3p	−0.136	0.959	−0.8	0.038	−1.034	0.038	−1.083	0.574
hsa-miR-30e	0.798	0.003	−0.048	0.881	1.107	0.010	0.466	0.065
hsa-miR-497	5.305	0.004	−0.143	0.302	5.1	0.011	4.304	0.001
hsa-miR-509-3p	−0.329	0.083	−0.06	0.653	0.825	0.234	−5.263	0.007
hsa-miR-559	−11.278	0.009	1.504	0.991	1.144	0.804	−7.323	0.007
hsa-miR-605	−3.301	0.164	4.458	0.233	−0.111	0.974	12.867	0.006
hsa-miR-609	−1.583	0.349	−0.832	0.613	−0.1	1.000	1.618	0.576
hsa-miR-610	−0.557	0.038	−0.463	0.291	0.493	0.382	−1.327	0.021
hsa-miR-632	−0.766	0.105	−0.495	0.350	0.176	0.721	−2.335	0.010
hsa-miR-645	−1.016	0.019	−0.628	0.135	−0.426	0.224	−5.643	0.001
hsa-miR-668	14.982	0.004	7.463	0.072	−2.096	0.549	11.805	0.017
hsa-miR-7	4.86	0.181	1.229	0.437	5.291	0.048	0.28	0.611
hsa-miR-768-3p	6.126	0.085	−7.483	0.206	−9.603	0.059	−9.625	0.059
hsa-miR-801	1.108	0.270	−0.261	0.787	−3.102	0.000	−6.163	0.008
**hsa-miR-923**	**−1.635**	**0.000**	**−1.184**	**0.001**	**−2.833**	**0.000**	**−6.284**	**0.007**
RNU24	11.491	0.000	9.167	0.000	1.901	0.291	3.178	0.089
RNU48	−3.073	0.074	0.595	0.649	−10.127	0.001	−9.95	0.000

miRNAs highlighted in bold were chosen for validation in the validation cohort of 97 plasma samples.

miRNAs were prioritised for subsequent confirmation in the validation sample set if they showed consistently altered levels between the control group and each of the disease groups, and if all or most of these changes were deemed to be statistically significant. MiR-34a was chosen for validation as it was increased in the plasma of diseased patients compared to controls. MiR-150 and miR-923 were chosen for validation as their plasma levels progressively decreased as the sample/disease groups progress toward malignancy (Table 2).

### Altered levels of miR-34a, miR-150, and miR-923 in the validation cohort

The three candidate miRNAs; miR-34a, miR-150, and miR-923 were analysed in a validation cohort of 97 independent plasma samples comprising 20 normal, 20 polyp, 20 adenoma samples, 23 early stage cancer samples, and 14 advanced cancer samples (see Table 3).

**Table 3:** **Fold changes and associated p-values for miR-34a, miR-150, and miR-923 in the validation cohort**

	miR-34a		miR-150		miR-923	
	FC	P value	FC	P value	FC	P value
Normal vs Polyp	−1.3	0.286	1.52	0.218	1.24	0.537
Normal vs Adenoma	**2.09**	**0.028**	1.68	0.160	1.19	0.537
Normal vs Early Stage Cancer	**2.84**	**0.002**	−1.03	0.800	1.19	0.675
Normal vs Advanced Cancer	1.8	0.081	−1.52	0.282	−1.7	0.537
Polyp vs Adenoma	**2.71**	**0.002**	1.11	0.735	−1.04	0.904
Polyp vs Early Stage Cancer	**3.69**	**0.000**	−1.57	0.160	−1.05	0.779
Polyp vs Advanced Cancer	**2.34**	**0.006**	**−2.31**	**0.007**	−2.11	0.113
Adenoma vs Early Stage Cancer	1.36	0.381	−1.73	0.104	−1.01	0.867
Adenoma vs Advanced Cancer	−1.16	0.691	**−2.55**	**0.001**	−2.03	0.113
Early Stage Cancer vs Advanced Cancer	−1.58	0.169	−1.47	0.529	−2.02	0.113
Non-Cancer vs Early Stage Cancer	**3.07**	**0.000**	1.42	0.160	1.19	0.675
Non-Cancer vs Cancer	**2.62**	**0.001**	**1.29**	**0.013**	−1.11	0.779
Normal & Polyp vs Adenoma	**1.9**	**0.002**	1.98	0.282	1.08	0.537

Bold text denotes significant comparisons. Non-Cancer comprises normal, polyp & adenoma groups, Cancer comprises both early stage & advanced cancer groups.

**Figure 1:**
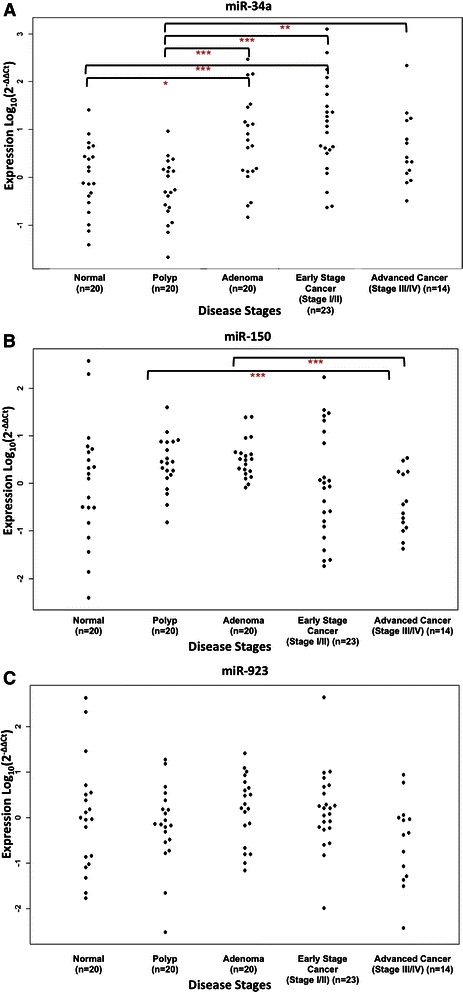
MiR-34a, miR-150, and miR-923 in the validation cohort. Beeswarm plots of plasma levels of **(A)** miR-34, **(B)** miR-150, and **(C)** miR-923 in the five sample groups; normal (n = 20), polyp (n = 20), adenoma (n = 20), early stage cancer (stage I/II) (n = 23), and advanced cancer (stage III/IV) (n = 14). Expression levels of the miRNAs (log_10_ scale at y-axis) are normalised to let-7e. Statistically significant differences between groups were determined using Wilcoxon rank sum tests and are represented as follows; p-value <0.05 = *, p-value <0.01 = **, p-value < 0.001 = ***.

Among the three miRNAs analysed in the validation cohort, only miR-34a distinguished the normal and precancerous lesion groups from the disease samples (Figure 1A). MiR-34a expression was significantly increased in the adenoma (FC 2.09, p-value = 0.028) and early stage cancer (FC 2.84, p-value = 0.002) groups compared to healthy controls, and moderately in the advanced cancer group (FC 1.80, p-value = 0.081). The levels of this miRNA were also significantly higher in the adenoma (FC 2.71, p-value = 0.002), early stage cancer (FC 3.69, p-value = 3.90e-5) and advanced cancer (FC 2.34, p-value = 0.006) groups compared to the polyp samples. Alternatively, miR-150 distinguished the polyp and adenoma groups from the advanced cancer ((FC -2.31, p-value = 0.007, FC -2.55, p-value = 0.001 respectively) (Figure 1B). Following adjustment for multiple testing, miR-923 was not found to have significantly altered levels in the validation cohort (Figure 1C).

### Validation of altered miR-34a, miR-150, and miR-923 expression in an independent dataset of matched colon tumour and normal tissues

In an effort to determine whether the levels of circulating cell free miRNAs that were observed in our study reflected the biology of the tumour, we investigated their expression in a publically available qPCR dataset of CRC tumours and matched normal tissues [21]. All miRNAs found to be significantly differentially expressed in the independent dataset are listed in Additional file 1. As this independent dataset only contains information on cancer and adjacent normal samples, the normal, polyp and adenoma plasma samples in our validation cohort were combined into a ‘non-malignant’ group and the early stage and advanced cancer plasma samples were combined into a ‘cancer’ group to facilitate comparison of the miRNA expression changes.

**Figure 2:**
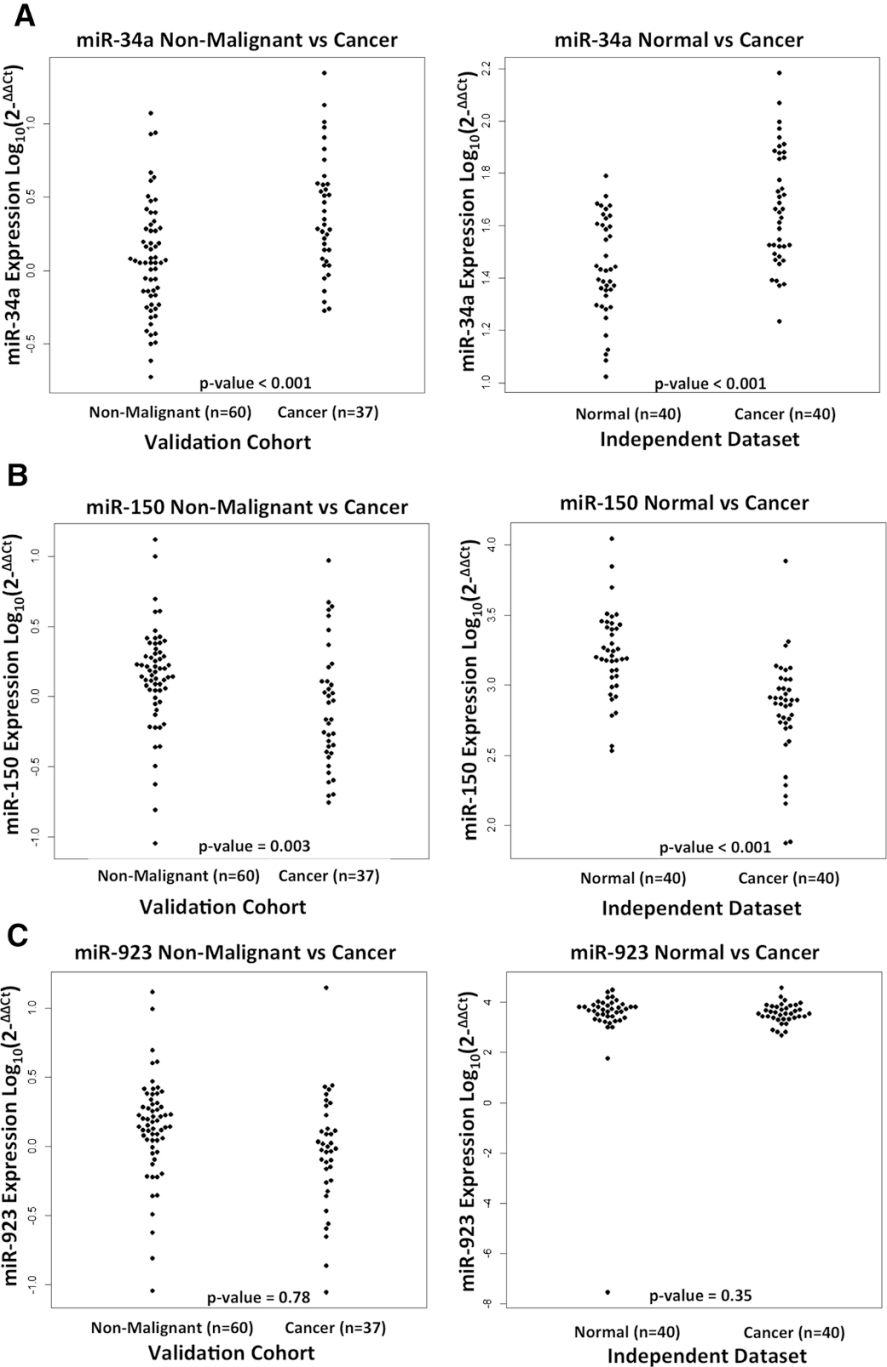
Expression levels of miR-34a, miR-150, and miR-923 in cancerous and non-malignant sample groups in the validation cohort and independent data set. **(A)** Cancer samples show significantly higher miR-34a expression (p-value < 0.001) compared to non-malignant samples in both the validation plasma samples and independent tumour tissue samples. **(B)** Cancer samples show significantly lower miR-150 expression compared to non-malignant samples in both the validation plasma samples (p-value =0.003) and independent tumour tissue samples (p-value < 0.001). **(C)** No significant change in miR-923 expression is observed in either the validation plasma samples (p-value = 0.56) or the independent tumour tissue samples (p-value = 0.35). The non-malignant group in the validation cohort represents the normal, polyp, and adenoma samples. Cancer groups in both studies represent cancers of all stages I-IV. Expression levels of the miRNAs (log_10_ scale at y-axis) are normalised to let-7e in the validation cohort and mean normalised in the independent dataset. Statistically significant differences between groups were determined using Wilcoxon rank sum tests in both the validation and independent cohorts.

Figure 2 illustrates that the altered levels of all three circulating miRNAs observed in the plasma samples of the validation cohort mirrors the expression changes in the CRC tissues in the independent tissue sample set. Mir-34a was found to be significantly up-regulated in both the validation and the independent sample sets, 2.62 fold up-regulated (p-value = <0.001) and 1.71 fold up-regulated (p-value = 8.50e-6) respectively (Figure 2A). In further examining the expression of miR-34a in the combined plasma sample groups in the validation cohort, it was observed that it was also able to distinguish the non-malignant group (normal, polyp & adenoma groups) from the early stage cancer group (FC = 3.07, p-value = <0.001), and the normal and polyp groups from the benign adenoma group (FC = 1.90, p-value = 0.001). Additionally, miR-150 was significantly down-regulated in both the cancer plasma (FC = -1.29, p-value = 0.013) and cancer tissue (FC = -2.55, p-value = 4.104e-08) samples. Although not significantly, miR-923 was also down-regulated in both the cancer plasma (FC = -1.11, p-value = 0.779) and cancer tissue (FC = -2.26, p-value = 0.353) samples. See Table 3 for all FCs and associated p-values for miR-34a, miR-150, and miR-923 in the validation cohort.

### Diagnostic potential of circulating miR-34a, miR-150, and miR-923 for the detection of disease of the colon

**Figure 3:**
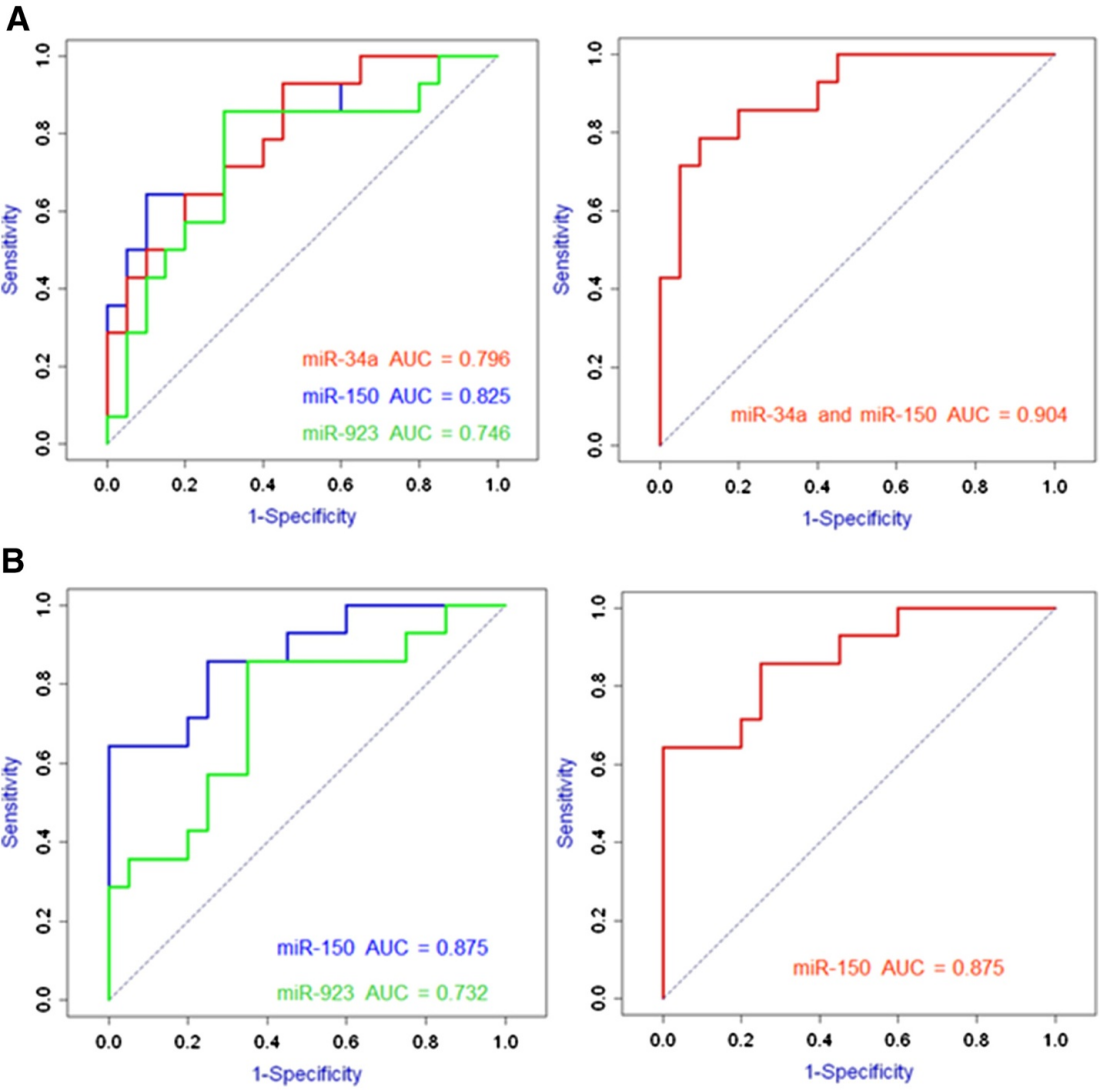
ROC curve analysis combining plasma levels of the three miRNAs to distinguish advanced cancer from benign disease groups. **(A)** Plasma levels of miR-34a, miR-150, and miR-923 discriminate polyp samples (n = 20) from advanced cancer (stage III/IV) samples (n = 14) individually; AUC = 0.796, 0.825, and 0.746 respectively. Combining markers miR-34a and miR-150 increases the AUC to 0.904. miR-923 expression does not improve the fit of the model and thus is not included in the combination. **(B)** Plasma levels of miR-150 and miR-923 discriminate adenoma samples (n = 20) from advanced cancer (stage III/IV) samples (n = 14) individually; AUC = 0.875 and 0.732 respectively. Combining these markers does not improve the fit of the model, thus miR-150 alone distinguishes adenoma samples from advanced cancer samples with an AUC of 0.875.

Once the altered expression of miR-34a, miR-150, and miR-923 was confirmed in our 97 validation plasma samples, and in the independent tissue samples, LR and ROC analyses were used to evaluate the potential of these miRNAs to distinguish between the disease and control blood plasma samples. First LR was used to identify the linear model with the best discriminatory power between sample groups, and the quality of this model was depicted by the area under the curve (AUC) of the ROC curve. For individual miRNAs, AUCs ranged from 0.488 to 0.875 (see Additional file 2). In an effort to identify the most powerful candidates as diagnostic markers of disease, we focused on comparing sample groups that showed significant differential expression of more than one of the three miRNAs. Thus we have identified sets of miRNAs that could distinguish advanced cancer from benign disease groups (Figure 3). Individually, plasma levels of miR-34a, miR-150, and miR-923 discriminate polyp samples from advanced cancer samples with AUC = 0.796 (CI:0.646-0.947), 0.825 (CI:0.681-0.969), and 0.746 (CI:0.412-0.817) respectively. The discriminatory power of the analysis was improved by combining markers miR-34a (p-value = 0.016, CI:-2.911- -0.314) and miR-150 (p-value = 0.031, CI:0.392-2.640) which increased the AUC to 0.904. The Akaike’s Information Criterion (AIC) was used as a measure of the quality of the model. The model with the lowest AIC was considered the best fit i.e. the combination of miRNAs that produced the lowest AIC. Where the addition of a miRNA did not lower the AIC it was excluded as the simplest model that best describes the data is preferable. MiR-923 expression was found not to improve the fit of the model and thus was not included in the combination (Figure 3A). In searching for an optimal model to distinguish adenoma samples from advanced cancer samples, it was found that miR-150 alone (rather than in combination with miR-923) was sufficient to with an AUC of 0.875 (CI:0.754-0.996) (Figure 3B).

## Discussion

Screening for the early detection of CRC is important to improve patient survival and facilitate cancer prevention through the detection and removal of polyps. The aim of this study therefore was to investigate the potential of circulating cell-free miRNAs not only as biomarkers of CRC, but also their efficiency at delineating patients presenting with precancerous lesions, i.e. polyps and benign adenomas from normal and cancer patient groups. MiRNA profiling was performed in the discovery sample cohort consisting of five groups; normal, polyps, benign adenomas, early stage cancer, and advanced cancer (Table 2) and identified three candidate miRNAs (miR-34a, miR-150, and miR-923) which were then further examined in a validation cohort of 97 samples divided into the same five groups as before (Figure 1 and Table 3). In addition, we confirmed that the altered circulating levels of miR-34a and miR-150 mirror the expression changes evaluated in the tumours of an independent dataset of 40 CRC samples and their paired normal tissues (Figure 2).

miR-34a is a p53-regulated miRNA that has been shown to influence both cellular senescence and apoptosis [24,25]. Different studies have demonstrated its up or down regulation in CRC compared to normal tissue (as reviewed in [26]). Wu *et al.* [27] demonstrated the involvement of this miRNA in CRC invasion and metastasis through targeting FRA1, a FOS transcription factor that is capable of forming activator protein-1 (AP-1) heterodimers. Increased levels of this circulating miRNA have been detected in patients with chronic hepatitis C and non-alcoholic fatty liver disease [28] and levels have been found to be decreased in whole blood samples of patients with CRC compared to healthy controls [29]. Brunet and colleagues studied miRNA expression in stage III CRC tissue samples compared to normal controls and found miR-34a to be significantly up-regulated [30].

In one of the first studies to investigate the altered expression of miRNAs in cancer [31] miR-150 was shown to be up-regulated in colorectal tissue compared to normal tissue. However several subsequent studies have shown this miRNA to be down-regulated in CRC tissue compared to normal tissue [21,32]. Indeed a recent study on 239 samples from Ma *et al.* found that miR-150 was down-regulated in adenoma and CRC tissues compared to normal tissue, and this down-regulation was associated with decreased overall survival and a worse response to adjuvant chemotherapy [33]. Decreased circulating levels of miR-150 have been identified in patients with acute myeloid leukaemia [34], and have been associated with poor prognosis for critically ill patients [35]. Furthermore, Wang and colleagues found miR-150 expression to be down-regulated in their 10 pooled CRC plasma samples compared to 10 pooled control samples, although its altered expression was not validated in their additional individual samples [15]. To our knowledge there are no studies outlining miR-923 expression in CRC or detecting circulating levels of this miRNA, however it has been shown to be down-regulated in chronic lymphocytic leukaemia patients [36] and up-regulated in taxol resistant breast cancer cells [37].

Of the three miRNAs analysed in our validation cohort, only miR-34a distinguished the normal group from the disease groups (Figure 1A). A large amount of inter-individual variability was noted in the normal samples assessed for miR-150 and miR-923 expression which may account for the fact that they do not significantly separate the normal group from the disease groups (Figure 1B&C). The reason for this variability may lie in the fact that these subjects had sufficient medical complaints to present themselves for colonoscopy, but although they do not present polyps, adenomas or cancer of the colon they may have had other conditions such as irritable bowel disease which may influence the results. In addition, the high number of adenoma samples (n = 16) compared to the other sample group numbers (n = 8) in the discovery cohort may explain why we observed statistically significant alterations in miR-150 and miR-923 in the initial analysis (Table 2) but not in the validation cohort (Table 3). Despite the variability within sample groups, we found circulating levels of miR-34a and miR-150 to be capable of distinguishing cancer patients from the non-malignant group of patients (Figure 2), in addition they were also capable of delineating patient groups with different diseases of the colon from each other (Table 3). Moreover, the discovery miRNA profiling results (Table 2) provide additional miRNA candidates, for instance miR-144-5p that may have potential as circulating miRNA biomarkers of CRC which can be exploited and independently validated by other research groups. In our opinion this is an important step towards the identification of specific biomarkers for early stages of disease.

There have been several publications examining the potential for miRNAs to act as circulating biomarkers for the detection of CRC. Recently, Faltejskova and colleagues attempted to validate the serum levels of four miRNAs (miR-17-3p, miR-29a, miR-92a and miR-135b) that had been proposed by other groups as potential circulating biomarkers of CRC. They used qPCR to assess the miRNA expression levels in 100 CRC patients and 30 healthy controls, and did not detect any significant changes in the expression of any of the miRNAs evaluated [17]. We examined the lists of significantly differentially expressed miRNAs in our discovery cohort to determine whether we also identified biomarkers found by other groups. We did not find miR-21 [19], miR-141 [38], miR-29a, miR-17-3p or miR-92 [14,16], miR-601 or miR-760 [15] to be significantly differentially expressed in any of the disease groups compared to our healthy controls. We did, however find miR-19a, miR-19b, and miR-15b significantly altered in some of our comparisons (see Table 2). These miRNAs were among those found by Giráldez *et al.* to be significantly up-regulated in plasma samples of CRC patients in their study in 2012 [18].

Although there is some concordance among the results of different groups in the search for biomarker miRNAs, uncertainty remains as to which miRNAs are the most appropriate markers of disease. Disparities in patient age and time of sample collection (i.e. before or after surgery/treatment) in different studies may impact on the reproducibility of results. In addition to these variables, it has been noted previously that miRNA profiles vary between different ethnic groups [39], male and female patients [40], and that blood cell contaminants can contribute to circulating miRNA profiles [40,41]. Blood cell contaminants of plasma and serum samples may be of particular importance in evaluating the potential of circulating miRNAs as biomarkers of disease. In fact, Pritchard and colleagues suggest that the elevated miR-92a levels detected in the plasma of patients with colon cancer are due the higher levels of red blood cell haemolysis in patients with this disease [41]. This poses the question as to whether all of these putative biomarkers should be discarded due to their expression in blood cells, or whether extensive validation and perhaps additional profiling on larger more diverse patient cohorts will confirm the most reliable biomarker candidates. We would argue against discarding biomarkers because of their detection in hematopoietic cells, particularly if, as we have shown, their expression reflects that found within the tumour (Figure 2). If we were to discard markers for their presence in blood cells, we would also have to discount miR-21 as a valid marker of disease as it was also detected by Duttagupta and colleagues [40]. This miRNA is commonly up-regulated in cancer, has been identified in the serum and stool samples of cancer patients, and multiple studies have linked its expression to advanced disease and worse outcome for patients [42]. In an effort to control confounding factors in this study, all samples were age and sex matched, blood was taken at the time of colonoscopy before treatment commenced, and an additional centrifugation step to remove cellular debris prior to RNA extraction recommended by Duttagupta and colleagues [40] was included in the sample processing.

In order to examine the diagnostic potential of our three candidate miRNAs in detecting different diseases of the colon we employed ROC curve analysis. To identify the most powerful candidate combinations we focused our analysis on comparing sample groups that showed significant differential expression of more than one of the three miRNAs. This approach allowed us to identify marker combinations which distinguish patient groups with benign disease of the colon from those with advanced stage cancer (Figure 3). Specifically, miR-34a and miR-150 abundance were capable of differentiating patients with polyps from those with advanced cancer, AUC = 0.904, and miR-150 abundance separates patients with adenomas from those with advanced cancer, AUC = 0.875. To further confirm the true diagnostic potential of these circulating cell-free miRNAs it is now important for these results to be independently replicated in additional samples by another group. If this independent validation were successful, a prospective validation of the miRNA candidate biomarkers would be warranted.

## Conclusions

To our knowledge this is the first study to examine circulating miRNA levels in samples from patients with polyps in addition to normal, adenoma, and early stage and advanced cancer samples. We identified two circulating miRNAs capable of distinguishing patient groups with different diseases of the colon from each other, and sets of miRNAs that distinguish patients with advanced cancer from benign disease groups. We also found miR-34a expression to be significantly increased in early stage cancer samples compared to the non-malignant patient samples, and in adenoma samples compared to normal and polyp samples.

## Additional files

Additional file 1:
**Significantly differentially expressed miRNAs in the independent dataset.**

Additional file 2:
**AUC values for miR-34a, miR-150, and miR-923 in the validation cohort.**

## Abbreviations

CRC: Colorectal cancer
LR: Logistic regression
ROC: Receiver operator curve
AUC: Area under the curve
FC: Fold change
